# Effect of a Mobile Phone Intervention on Quitting Smoking in a Young Adult Population of Smokers: Randomized Controlled Trial

**DOI:** 10.2196/10893

**Published:** 2018-10-23

**Authors:** Neill Bruce Baskerville, Laura Louise Struik, Godefroy Emmanuel Guindon, Cameron D Norman, Robyn Whittaker, Catherine Burns, David Hammond, Darly Dash, K Stephen Brown

**Affiliations:** 1 Propel Centre for Population Health Impact Faculty of Applied Health Sciences University of Waterloo Waterloo, ON Canada; 2 School of Public Health and Health Systems Faculty of Applied Health Sciences University of Waterloo Waterloo, ON Canada; 3 School of Pharmacy Faculty of Science University of Waterloo Kitchener, ON Canada; 4 Centre for Health Economics and Policy Analysis Department of Health Research Methods, Evidence, and Impact McMaster University Hamilton, ON Canada; 5 Cense Ltd Toronto, ON Canada; 6 Dalla Lana School of Public Health University of Toronto Toronto, ON Canada; 7 National Institute of Health Innovation University of Auckland Auckland New Zealand; 8 Systems Design Engineering University of Waterloo Waterloo, ON Canada; 9 Department of Health Research Methods, Evidence, and Impact McMaster University Hamilton, ON Canada; 10 Statistics and Actuarial Science University of Waterloo Waterloo, ON Canada

**Keywords:** health behavior, smoking cessation, young adult, mobile phone, mHealth, randomized controlled trial

## Abstract

**Background:**

Digital mobile technology presents a promising medium for reaching young adults with smoking cessation interventions because they are the heaviest users of this technology.

**Objective:**

The aim of this study was to determine the efficacy of an evidence-informed smartphone app for smoking cessation, Crush the Crave (CTC), on reducing smoking prevalence among young adult smokers in comparison with an evidence-informed self-help guide, On the Road to Quitting (OnRQ).

**Methods:**

A parallel, double-blind, randomized controlled trial with 2 arms was conducted in Canada to evaluate CTC. In total, 1599 young adult smokers (aged 19 to 29 years) intending to quit smoking in the next 30 days were recruited online and randomized to receive CTC or the control condition OnRQ for a period of 6 months. The primary outcome measure was self-reported continuous abstinence at the 6-month follow-up.

**Results:**

Overall follow-up rates were 57.41% (918/1599) and 60.48% (967/1599) at 3 and 6 months, respectively. Moreover, 45.34% (725/1599) of participants completed baseline, 3-, and 6-month follow-up. Intention-to-treat analysis (last observation carried forward) showed that continuous abstinence (N=1599) at 6 months was not significantly different at 7.8% (64/820) for CTC versus 9.2% (72/779) for OnRQ (odds ratio; OR 0.83, 95% CI 0.59-1.18). Similarly, 30-day point prevalence abstinence at 6 months was not significantly different at 14.4% (118/820) and 16.9% (132/779) for CTC and OnRQ, respectively (OR 0.82, 95% CI 0.63-1.08). However, these rates of abstinence were favorable compared with unassisted 30-day quit rates of 11.5% among young adults. Secondary measures of quit attempts and the number of cigarettes smoked per day at 6-month follow-up did not reveal any significant differences between groups. For those who completed the 6-month follow-up, 85.1% (359/422) of young adult smokers downloaded CTC as compared with 81.8% (346/423) of OnRQ, χ^2^_1(N=845)_=1.6, *P*=.23. Furthermore, OnRQ participants reported significantly higher levels of overall satisfaction (mean 3.3 [SD 1.1] vs mean 2.6 [SD 1.3]; *t*_644_=6.87, *P*<.001), perceived helpfulness (mean 5.8 [SD 2.4] vs mean 4.3 [SD 2.6], *t*_657_=8.0, *P*<.001), and frequency of use (mean 3.6 [SD 1.2] vs mean 3.2 [SD 1.1], *t*_683_=5.7, *P*<.001) compared with CTC participants.

**Conclusions:**

CTC was feasible for delivering cessation support but was not superior to a self-help guide in helping motivated young adults to quit smoking. CTC will benefit from further formative research to address satisfaction and usage. As smartphone apps may not serve as useful alternatives to printed self-help guides, there is a need to conduct further research to understand how digital mobile technology smoking cessation interventions for smoking cessation can be improved.

**Trial Registration:**

ClinicalTrials.gov NCT01983150; http://clinicaltrials.gov/ct2/show/NCT01983150 (Archived by WebCite at http://www.webcitation.org/6VGyc0W0i)

## Introduction

Tobacco use among young adults remains a global public health issue as young adults continue to maintain high prevalence rates [1]. For example, compared with the national average of 13%, the prevalence of smoking among young adults in Canada was 18.5% among those aged 20 to 24 years in 2015 [2]. Globally, smoking is responsible for taking approximately 6 million lives and costing about US $500 billion per year [3]. However, quitting before the age of 40 years significantly reduces the morbidity and mortality rates related to smoking [4], making young adults a priority for smoking cessation efforts.

Younger age groups have the highest quit attempt rates, which decline with age [2], indicating that young adults are a ripe audience for assisting in smoking cessation. However, young adults are reported to not use cessation interventions, including pharmacological or psychological treatments [5-7], compared with their older counterparts. The lack of tailored cessation interventions for this age demographic has been cited as a major reason for this underutilization [7]. In addition, the personal and societal values of independence and autonomy may influence the general trend of unassisted smoking cessation among young adults [2,8,9].

A promising new direction for reaching and engaging young adults in smoking cessation interventions is the use of mobile phones, particularly smartphone apps. Smartphone ownership among young adults aged 18 to 34 years in both the United States and Canada is almost ubiquitous at 92% and 94%, respectively [10]. It is not surprising then that young adults lead the way in downloading and using health apps [11] and are the most frequent users of health-related apps [12].

The advanced processing capabilities, global reach, and unmatched accessibility of smartphones render them ideal channels for delivering health-related interventions [13]. In addition, the complex functionalities enabled in smartphone apps facilitate high user engagement, which is a strong predictor of smoking cessation [14]. Encouragingly, smartphone apps have shown to be particularly appealing to young adults for receiving cessation support [15]. In light of their growing popularity, many cessation apps are now available [14,16,17]. However, very few are based on evidence or theory or have been rigorously evaluated [18,19].

Although there is a growing body of evaluative evidence on the efficacy and effectiveness of smartphone-based technologies for smoking cessation, this has largely been through small pilot studies. Although most evaluative evidence consists of studies of short message service (SMS) text-messaging-based interventions for smoking cessation [20,21], the body of evidence in relation to apps specifically is nascent. Two recent systematic reviews focused on smartphone apps for smoking cessation. Haskins et al identified 7 studies of smoking cessation apps and searched 177 unique smoking cessation apps on the iTunes app store and 139 unique smoking cessation apps on Google Play. They concluded that of the top 50 apps from these leading app stores, only 2 had any published scientific support [22].

A systematic review of 8 studies found mixed evidence regarding the effectiveness of smoking cessation apps and observed that user engagement and adherence to app features influenced quit rates and that larger sample size studies are needed [23]. Two of the apps examined were supported by small randomized controlled trials (RCTs) [14,24], and 1 was an observational study [25]. Only 1 small study specifically targeted young adults aged 18 to 30 years, and the authors found that the smartphone app did not move smokers to quit as quickly as SMS text messaging [24]. Another pilot study with older adults tested the efficacy of a smoking cessation app based on acceptance and commitment therapy and found that it was feasible to deliver a theory-based smartphone app; however, the quit rates were not significantly different between conditions [14]. The third small observational study tested a smoking cessation app based on behavior change theory and found that participants were more likely to be abstinent from smoking for 28 days or longer as compared with the general smoking population [25].

One recent RCT examined the effect of an evidence-informed decision-aid app on continuous abstinence at 1, 3, and 6 months among adults aged 18 years and older who were motivated to quit [26]. The authors found that the decision-aid app, based on the Ottawa Decision Support Framework, significantly predicted abstinence at all 3 time points compared with the control app, which did not provide any structured process for considering options, benefits and harms of quitting methods, and ongoing support of a decision [26].

Our aim was to conduct a large and methodologically rigorous evaluation of smartphone cessation technology to address the identified gap in the published literature. We compared the effects of an evidence-informed smoking cessation mobile phone app known as Crush the Crave (CTC) with a self-help booklet on reducing smoking prevalence among young adult smokers after 6 months. The mobile phone app was hypothesized to yield higher rates of continuous abstinence, 30-day point prevalence abstinence (PPA), 7-day PPA, quit attempts, and reduction in consumption of cigarettes. This is the first full-scale and long-term study that we are aware of to assess the efficacy of a quit smoking app that specifically targets young adults.

## Methods

### Study Design

**Figure 1:**
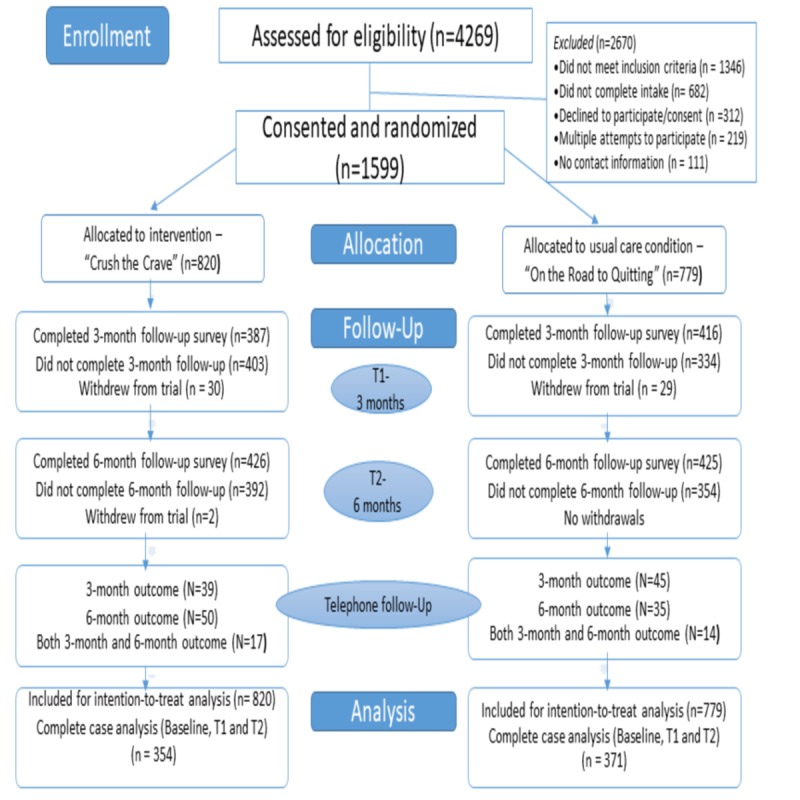
Consolidated Standards of Reporting Trials (CONSORT)-EHEALTH diagram.

The study was a 6-month, 2-arm, parallel RCT conducted in Canada with participants assigned with an equal probability to the mHealth intervention, CTC, or to the self-help booklet. Investigators, data collectors, and participants were blinded to the group assignments. Follow-up was conducted at 3- and 6-months post randomization. A superiority trial design was used [27] and the protocol was in accordance with the Consolidated Standards of Reporting Trials (CONSORT)- EHEALTH checklist [28]. See Figure 1 for a CONSORT diagram of the proposed study design. A complete description of the study protocol has been published elsewhere [29].

### Participants and Recruitment

Participants were eligible if they were between the ages of 19 and 29 years, smoked cigarettes daily, resided in Canada, were considering quitting smoking in the next 30 days, had an Android (Version 2.0 to 5.0) or iPhone (Version 4.0 to 7.0) smartphone, were able to provide informed consent, were able to comprehend English, and were not referred to the study by an existing study participant—a friend or family member already participating in the study—to avoid possible contamination bias.

Recruitment sources were primarily Web-based media and included 72% from Facebook advertisements, 24% Google advertisements, and 4% from other sources (a Web-based classified message board and offline recruitment through a commercial survey panel). Interested young adults were referred to a website describing the study. Potential participants were screened at the entry webpage where their eligibility was determined, informed consent was sought, and registration for the study conducted. Participants who met the inclusion criteria and consented to participate completed the Web-based baseline questionnaire and were then randomly allocated to either the control or intervention arm and were sent a computer-generated email confirming registration and containing instructions for downloading their randomly assigned quit smoking program (see Multimedia Appendix 1). Participants were sent a reminder email at 1 month after completing the baseline questionnaire to download the assigned program if they had not already done so. Participants were provided a Can $35 incentive for registering to the study, and a raffle of an iPad Air tablet was used as an incentive to complete 6-month follow-up.

### Interventions

#### Crush the Crave

The intervention group received a comprehensive and evidence-informed smoking cessation smartphone app, CTC version 2.1. CTC was developed in early 2012 by a team of population health researchers, social media experts, and computer programmers as an evidence-informed quit smoking smartphone and social media app for young adults aged 19 to 29 years [29]. CTC enabled users to customize a quit plan by choosing a quit date and then deciding whether to quit immediately or reduce the number of cigarettes they smoke every week up to their quit date. CTC then assisted smokers in staying on track by reminding them of how much money they had saved and how much their health had improved over time after quitting. On the basis of contingency reinforcement, milestones were tracked as rewards, which smokers could choose to share with their social network via Facebook and Twitter, and rally support from friends and family. Participants could also link to the CTC Facebook community for additional support for quitting. Users of the app received supportive messages and inspirational photos via the app tailored to their specific quit plan and where they were in the quitting experience. Participants could also record when, where, and why they were smoking to understand the triggers and psychosocial factors related to smoking. The app provided both graphic and tabular performance feedback; Web-based distractions to help deal with cravings; evidence-informed information for assisting smokers on topics such as relapse and dealing with cravings, push notifications on rewards received, and helpful reminders to continue to use the app; and access to evidence-based cessation services such as smoking cessation quitlines and information on the use of nicotine replacement therapy (NRT).

Recently, Ubhi et al conducted a review of 137 smoking cessation apps for the presence or absence of evidence-based behavior change techniques and CTC addressed 4 out of 5 behavior change strategies as compared with an average of only 1 across the 137 apps reviewed. They also assessed CTC as having an ease-of-use score of 95%, which was similar to the average of all apps reviewed and 82% for engagement compared with only 45% overall [30]. A more fulsome description of the evidence for and development of the CTC app is available elsewhere [31]. The development of CTC was frozen during the study.

#### Self-Help Booklet

The control group received a standard print-based self-help guide known as On the Road to Quitting (OnRQ) [32] that was developed by Health Canada for young adult smokers. Participants were able to both view and download the self-help guide via the internet and request a printed version of the guide. Moreover, 47.0% (366/779) of participants allocated to the control group requested a printed version of the guide. The guide contained similar content to the CTC app. It contained information on the health benefits of quitting, the monetary rewards of quitting, smoking triggers, suggestions on how to deal with withdrawal and cravings, setting a quit date or quitting *cold turkey*, seeking counseling or NRT, linking to a social support network of family or friends, telephone quitline support, preventing weight gain, and dealing with slip-ups or relapse.

### Study Procedures

All study procedures were reviewed and approved by the clinical research ethics review committee of the University of Waterloo (Full ethics clearance October 29, 2013, 19275).

### Randomization and Blinding

Participants had an equal probability of being allocated to the intervention or control group using a computer-generated simple randomization procedure. Participants were blinded to group allocation and were not aware of which was the control and intervention condition. Investigators were blinded to group allocation until completion of the trial after initial analysis of the primary and secondary outcomes.

### Data Collection

Baseline data were collected via a self-administered and Web-based questionnaire completed by all participants who provided Web-based consent to participate in the study for both intervention and control groups from July 2014 to March 2015. The baseline questionnaire included the following demographic items: age, gender, ethnicity, marital status, education, income, and employment status. Variables related to tobacco consumption included current smoking status, amount smoked, number and duration of past quit attempts, and the degree of nicotine dependence. Participants were also asked a series of psychosocial questions, including beliefs and attitudes about quitting, self-efficacy or confidence in quitting, perceived stress and social support, and social norms related to smoking. Furthermore, participants were asked about the experience with smartphone apps and self-help, use of NRT, e-cigarette use, and other cessation aid or supports, such as quitlines.

Follow-up data were collected from the same participants at 3- and 6-months postrandomization in the same manner as the baseline. In addition to the questions asked at baseline, participants were asked core smoking status questions. Participants were also asked questions on nicotine withdrawal, level of support received from friends and family for quitting smoking, use of e-cigarettes, additional cessation services that they sought for help to quit, overall satisfaction with the app or self-help guide, use of and opinions and beliefs about the app and the guide, and challenges they experienced in quitting smoking. All instruments were piloted with a convenience sample (n=10) that comprised an equal number of male and female young adult smokers. A modified Dillman method [33] was used for the follow-up of participants completing the Web-based survey questionnaires. Participants who did not complete 3- or 6-month follow-up questionnaires within 2 weeks were contacted by telephone and up to 10 attempts (email or phone) were made to collect smoking status at 3-month and 6-month follow-up or both. Following the intention-to-treat principle, participants were analyzed in the groups to which they were allocated, regardless of whether they received or adhered to the allocated intervention [34].

#### Measures

The primary outcome measure was continuous self-reported abstinence defined as having been abstinent for 3-months post baseline [35]. Secondary outcome measures were self-reported 30-day PPA from smoking at 3 and 6 months, operationalized as not having smoked any cigarettes, even a puff, or used other tobacco in the last 30-days [36]; 7-day PPA at 3 and 6 months [36]; the number of quit attempts—“How many times did you stop using tobacco for 24 hours or longer?”—over the past 3 and 6 months [37]; and the reduction in consumption of cigarettes at 3 and 6 months (“On average, how many cigarettes do you smoke per day on the days that you smoke”) [36]. Biochemical validation of smoking status was not performed.

Secondary measures included nicotine dependence using the 2-item Heaviness of Smoking Index (HSI) from the Fagerstrom Test for Nicotine Dependence that combines the number of cigarettes smoked per day and the time to the first cigarette in the morning [38]. High scores on the HSI indicate higher levels of addiction and greater difficulty in quitting. HSI was categorized as low (scores of 0-2), medium (scores of 3 and 4), and high (scores of 5 and 6). Self-confidence in quitting was measured using a 5-item Likert scale from 1 (*not at all*) to 5 (*extremely*) and the question, “How confident were you in your ability to quit smoking?” [39]. Perceived stress was measured with 4 question items on feelings of control and the ability to handle personal problems using 5-item Likert scales of 0 (*never*) to 4 (*very often*) and totaled to create a score [40]. Stress was categorized as low (scores of 0-6), medium (scores of 7 to 9), and high (scores 10 to 16). Current and past use of NRT and e-cigarettes were measured with the question, “Did you use or are you currently using NRT/e-cigarettes to help you quit smoking?” with a yes or no response option to current use and past use. A partner who smokes was measured by asking the question, “Does your partner, spouse, or significant other currently smoke?” and the number of friends smoking was measured by asking, “Of the five closest friends or acquaintances that you spend time with on a regular basis, how many of them are smokers?” Social support was measured with 3 question items on feelings of support from family and friends using 5-item Likert scales of 1 (*not at all*) to 5 (*extremely*) and totaled to create a score [39]. Support was categorized as low (scores of 3-8), medium (scores of 9-11), and high (scores 12-15).

Process measures included having downloaded the intervention and measures of use, satisfaction, and helpfulness at 3 and 6 months. Participants completed a brief satisfaction instrument that included 4 5-point Likert scale items such as “I used the program frequently” and “I thought the program was easy to use,” with response choices that ranged from *strongly agree* to *strongly disagree* [41]. Perceived overall satisfaction was measured on a 5-point Likert scale that ranged from *not at all satisfied* to *very satisfied* and helpfulness was measured on a 10-point scale that ranged from *not at all helpful* to *very helpful*.

#### Sample Size

Sample size calculations were based on the difference in the objective measure of the primary outcome event—continuous abstinence from smoking—between intervention and control groups. Assuming a ratio of 1:1 for intervention to control subjects, an alpha of .05, power of 80%, and an effect size equal to 10% in the intervention versus 6% in the control condition for self-reported continuous abstinence, the required sample size was 800 per group for a total of 1600 participants using a 2-tailed test of proportions [42].

#### Statistical Analysis

Demographic and smoking characteristics (eg, HSI, use of e-cigarettes, and self-confidence to quit) were compared between groups at baseline using a chi-square test of association or a Fisher exact test for binary variables. All participants were analyzed in the study arm to which they were randomized.

Logistic regression was used to test between-group comparisons on the primary outcome variable—continuous abstinence—and the secondary outcomes 7-day and 30-day PPA at 3- and 6-month follow-up. For comparisons of secondary continuous outcomes (number of quit attempts and cigarettes per day) that did not meet normal distribution assumptions, a nonparametric Mann-Whitney test was conducted. For the process measures of having downloaded the intervention, frequency of use, satisfaction and helpfulness, a chi-square test of association was applied to binary and categorical variables, and for the ordinal variables approximating a normal distribution, a *t* test for independent groups was applied.

The intention-to-treat principle was followed for the analysis of continuous abstinence and the outcomes 30-day and 7-day PPA using 3 approaches to handle missing information about smoking status: (1) imputation using the baseline observation carried forward or classifying nonresponders at 3 and 6 months as smokers in accordance with the Russel standard [35]; (2) imputation using the last observation of smoking status carried forward for nonresponders at 6 months; and (3) multiple imputation (n=18) by chained equations which used the observed predictors of outcome and the predictors of lost to follow-up to impute missing outcome data to correct for any potential bias caused by missing data [43]. The imputation model included age, sex, education, province, marital status, ethnicity, income, heaviness of smoking, self-efficacy, perceived stress, and intervention group. In addition, a complete case analysis was performed in which any participant with missing information on any outcome was excluded. Finally, a subgroup analysis was undertaken for key demographics, smoking and cessation characteristics, social support, and use of intervention variables to assess homogeneity in treatment effects using logistic regression. Tests were 2-sided, and statistical analyses were completed using SAS software version 9.4 (SAS Institute Inc, Cary, NC, USA).

## Results

### Participant Characteristics

Participants were enrolled from July 4, 2014 to March 31, 2015. Follow-up was completed in October 2015. As shown in Figure 1, a total of 4269 completed the Web-based screening survey, of whom 2670 were excluded because they did not meet the inclusion criteria, did not consent to participate, did not complete the baseline intake survey, or provided no contact information. Participants with repeat log-ins from the same IP address were recorded and excluded. In total, 1599 young adult smokers were eligible and consented to participate. Participants were randomly allocated to the CTC intervention condition (n=820) or to the OnRQ self-help control condition (n=779). The survey follow-up rates were 57.41% (918/1599) and 60.48% (967/1599) at 3 and 6 months, respectively. Moreover, 45.34% (725/1599) of participants were considered complete cases having completed baseline intake, 3-, and 6-month follow-up for the primary outcome (see Figure 1), without any significant difference between the intervention and control conditions in complete case follow-up proportions (43.2% vs 47.6%, χ^2^_1(N=1599)_=3.2, *P*=.08).

The intervention and control groups were balanced with regard to demographic, behavioral, and social support characteristics at baseline as well as at 6-month follow-up (see Table 1). Furthermore, there were no significant differences between conditions among participants lost to follow-up, confirming no apparent differential attrition between conditions [44]. Overall, the majority of participants were male (54.06%, 858/1587), white (75.06%, 1168/1556), had postsecondary education or higher (55.22%, 878/1590), and had incomes of less than CAD $45,000 (65.12%, 941/1445). At baseline, 26.81% (424/1581) of participants had moderate to high nicotine dependence and 25.67% (408/1589) smoked a pack of cigarettes per day or more. In addition, 52.72% (843/1599) were currently using or had used NRT in the past, and 60.79% (972/1599) were currently using or had used e-cigarettes in the past. Moreover, 32.17% (478/1486) reported a high level of social support at baseline whereas 84.68% (1321/1560) reported having 2 or more friends who smoked and 28.69% (457/1593) reported living with a partner who smokes (see Table 1).

### Smoking Cessation

Table 2 shows the primary and secondary smoking cessation outcomes after 3 and 6 months for intention-to-treat (n=1599) and complete cases (n=725). Intention-to-treat (baseline observation carried forward) continuous abstinence after 6 months was not significantly different at 6.1% (50/820) for CTC versus 7.3% (60/779) for OnRQ (odds ratio; OR 0.81, 95% CI 0.54-1.20, *P*=.28). Last observation carried forward was also not statistically significant for continuous abstinence at 7.8% (64/820) for CTC versus 9.2% (72/779) for OnRQ (OR 0.83, 95% CI 0.59-1.18, *P*=.30). Similarly, 30-day PPA at 6 months was not significantly different for baseline observation carried forward at 12.9% (106/820) and 15.8% (123/779, OR 0.79, 95% CI 0.60-1.05, *P*=.10) and for last observation carried forward at 14.4% (114/820) and 16.9% (132/779, OR 0.82, 95% CI 0.63-1.08, *P*=.16) for CTC and OnRQ participants, respectively. However, 7-day PPA at 6 months using baseline observation carried forward showed a significant difference in favor of OnRQ at 22.3% (174/779) versus 18.3% (150/820) for CTC (OR 0.79, 95% CI 0.61-0.99, *P*=.05). The significant difference for 7-day PPA at 6 months was not apparent with last observation carried forward at 22% (180/820) and 24.4% (190/779) for CTC and OnRQ participants, respectively (OR 0.87, 95% CI 0.69-1.10, *P*=.25).

Intention-to-treat continuous abstinence using multiple imputation analysis [43] to impute status for lost to follow-up participants was 12.6% (103/820) for CTC and 12.1% (94/779) for OnRQ participants (OR 1.05, 95% CI 0.78-1.41, *P*=.76). Intention-to-treat analysis based on multiple imputations for 7-day and 30-day PPA at 6 months showed no significant differences between conditions (see Table 2).

Findings for complete cases (n=725) showed continuous abstinence rates of 13.8% (49/354) for CTC and 15.4% (57/371) for OnRQ (OR 0.89, 95% CI 0.59-1.34, *P*=.56). For self-reported 7-day and 30-day PPA at 6 months findings were similar to the multiple imputation intention-to-treat analysis (see Table 2). At 3 months, no statistically significant differences were observed between CTC and OnRQ for 7-day and 30-day PPA according to the complete cases and the intention-to-treat analyses (see Table 2).

Secondary measures of quit attempts and the number of cigarettes smoked per day at 6-month follow-up for those who had not quit smoking at 6 months (n=671) did not reveal any significant difference between the groups. Moreover, 94.3% (249/264) of OnRQ participants and 91.4% (265/290) of CTC participants had made at least 1 quit attempt (χ^2^_1(N=554)_=1.8, *P*=.19]). Furthermore, there was no difference in the number of quit attempts between groups (Median=4, *P*=.82) and in the number cigarettes smoked per day between groups (Median=5 for OnRQ vs Median=6 for CTC, *P*=.44).

**Table 1:** Baseline characteristics of participants randomized to each arm, those lost to follow-up, and those remaining at 6 months.

Baseline variable		All participants			Participants lost to follow-up			Remaining participants		
		CTC^a^ (n=820), n (%)	OnRQ^b^ (n=779), n (%)	*P* value	CTC (n=394), n (%)	OnRQ (n=354), n (%)	*P* value	CTC (n=426), n (%)	OnRQ (n=425), n (%)	*P* value
**Demographics**										
	Female	364 (44.9)	365 (47.0)	.39	172 (44.3)	160 (45.2)	.81	192 (45.4)	205 (48.6)	.35
	Age 19 to 23 years	409 (49.9)	376 (48.3)	.52	206 (52.3)	182 (51.4)	.81	203 (47.7)	194 (45.7)	.56
	Single–never legally married	508 (62.6)	486 (62.8)	.92	242 (62.2)	213 (61.0)	.74	266 (62.9)	273 (64.2)	.68
	High school or less education	351 (43.0)	361 (46.6)	.15	183 (46.7)	181 (51.6)	.18	168 (39.6)	180 (42.6)	.39
	White	599 (75.4)	569 (74.8)	.79	285 (74.6)	265 (76.6)	.53	314 (76.0)	304 (73.3)	.36
	Paid work	539 (67.5)	527 (69.6)	.38	259 (67.5)	242 (70.4)	.50	280 (67.6)	285 (69.0)	.67
	Income < CAD $45,000	484 (65.6)	457 (64.6)	.71	249 (69.4)	213 (67.0)	.51	235 (62.0)	244 (62.7)	.84
**Smoking and quitting behavior**										
	Moderate to high nicotine dependence	219 (27.1)	205 (26.5)	.79	111 (28.7)	97 (27.6)	.73	108 (25.7)	108 (25.7)	.99
	Smokes at least a pack per day or more	210 (25.7)	200 (25.7)	.99	109 (27.8)	97 (27.4)	.90	101 (23.8)	103 (24.4)	.86
	Very or extremely confident to quit	330 (40.8)	302 (39.2)	.52	162 (41.7)	133 (37.9)	.30	168 (40.0)	169 (40.3)	.92
	High stress level	240 (30.5)	237 (31.7)	.60	123 (32.4)	104 (30.6)	.61	117 (28.7)	133 (32.6)	.22
	Used NRT^c^ currently or in the past	424 (51.7)	419 (53.8)	.41	196 (49.8)	169 (47.7)	.58	228 (53.5)	250 (58.8)	.12
	Used e-cigarettes currently or in the past	500 (61.0)	472 (60.6)	.87	242 (61.4)	210 (59.3)	.56	258 (60.6)	262 (61.7)	.75
**Friends or partner smoking and level of support**										
	Two or more close friends smoke	682 (85.1)	639 (84.2)	.60	331 (86.0)	287 (83.7)	.39	351 (84.4)	352 (84.6)	.92
	Living with partner who smokes	228 (28.0)	229 (29.4)	.52	106 (27.2)	107 (30.2)	.36	122 (28.7)	122 (28.8)	.98
	High social support level	235 (31.1)	243 (33.2)	.38	110 (30.7)	110 (33.1)	.50	125 (31.5)	133 (33.3)	.58

^a^CTC: Crush the Crave.

^b^OnRQ: On the Road to Quitting.

^c^NRT: nicotine replacement therapy.

**Table 2:** Comparison of Crush the Crave and On the Road to Quitting on primary and secondary outcomes at 3 and 6 months (intention-to-treat).

Outcomes			CTC^a^ (N=820)	OnRQ^b^ (N=779)	Odds ratio (95% CI)	*P* value
**Continuous self-reported abstinence at 6 months, n (%)**						
	Complete cases (n=725)		49 (13.8)^c^	5 (15.4)^d^	0.89 (0.59-1.34)	.56
	ITT^e^–baseline observation carried forward		50 (6.0)	60 (7.3)	0.81 (0.54-1.20)	.28
	ITT–last observation carried forward		64 (7.8)	72 (9.2)	0.83 (0.59-1.18)	.30
	ITT–multiple imputation of outcomes^f^		103 (12.6)	94 (12.1)	1.05 (0.78-1.41)	.76
**Secondary outcomes (3 months)**						
	**Self-reported non-smoking in past 7 days, n (%)**					
		Complete cases (n=708)^g^	105 (30.4)^h^	107 (29.5)^i^	1.05 (0.76-1.44)	.78
		ITT–baseline observation carried forward	133 (16.2)	124 (15.9)	1.02 (0.78-1.34)	.87
		ITT–multiple imputation of outcomes	268 (32.7)	243 (31.2)	1.07 (0.87-1.32)	.52
	**Self-reported non-smoking in past 30 days, n (%)**					
		Complete cases (n=712)^g^	61 (17.6)^j^	61 (16.7)^k^	1.06 (0.72-1.57)	.76
		ITT–baseline observation carried forward	72 (8.8)	71 (9.1)	0.96 (0.68-1.35)	.82
		ITT–multiple imputation of outcomes	151 (18.4)	146 (18.7)	0.98 (0.76-1.26)	.87
**Secondary outcomes (6 months)**						
	**Self-reported non-smoking in past 7 days, n (%)**					
		Complete cases (n=708)^g^	114 (33.3)^l^	143 (39.1)^m^	0.78 (0.57-1.06)	.11
		ITT–baseline observation carried forward	150 (18.3)	174 (22.3)	0.79 (0.61-0.99)	.05
		ITT–last observation carried forward	180 (22.0)	190 (24.4)	0.87 (0.69-1.10)	.25
		ITT–multiple imputation of outcomes	295 (36.1)	302 (38.8)	0.89 (0.73-1.09)	.27
	**Self-reported non-smoking in past 30 days, n (%)**					
		Complete cases (n=709 )^g^	84 (24.4)^n^	107 (29.3)^o^	0.78 (0.56-1.09)	.14
		ITT–baseline observation carried forward	106 (12.9)	123 (15.8)	0.79 (0.60-1.05)	.10
		ITT–last observation carried forward	114 (14.4)	132 (16.9)	0.82 (0.63-1.08)	.16
		ITT–multiple imputation of outcomes	199 (24.3)	220 (28.2)	0.81 (0.65-1.02)	.07

^a^CTC: Crush the Crave.

^b^OnRQ: On the Road to Quitting.

^c^N=354 for CTC for cases with continuous self-reported abstinence at 6 months.

^d^N=371 for OnRQ for cases with continuous self-reported abstinence at 6 months.

^e^ITT: intention-to-treat.

^f^Multiple imputation by chained equations (number of imputations=18).

^g^Number of cases is less than 725 because of missing data.

^h^N=345 for CTC for cases with self-reported non-smoking in past 7 days (secondary outcome at 3 months).

^i^N=363 for OnRQ for cases with self-reported non-smoking in past 7 days (secondary outcome at 3 months).

^j^N=347 for CTC for cases with self-reported non-smoking in past 30 days (secondary outcome at 3 months).

^k^N=365 for OnRQ for cases with self-reported non-smoking in past 30 days (secondary outcome at 3 months).

^l^N=342 for CTC for cases with self-reported non-smoking in past 7 days (secondary outcome at 6 months).

^m^N=366 for OnRQ for cases with self-reported non-smoking in past 7 days (secondary outcome at 6 months).

^n^N=344 for CTC for cases with self-reported non-smoking in past 30 days (secondary outcome at 6 months).

^o^N=365 for OnRQ for cases with self-reported non-smoking in past 30 days days (secondary outcome at 6 months).

**Figure 2:**
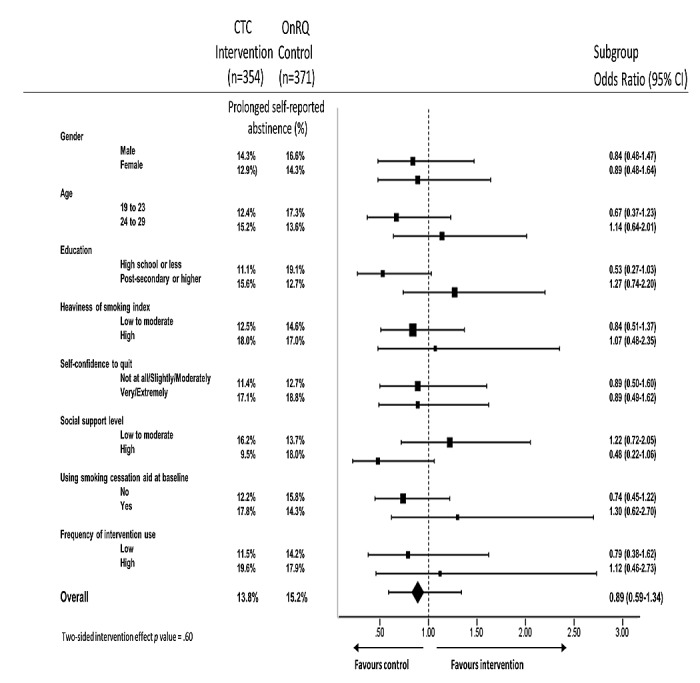
Effect of the Crush the Crave intervention on primary outcome by subgroup (N=725). CTC: Crush the Crave; OnRQ: On the Road to Quitting.

### Subgroup Analysis

For complete cases (N=725), the sub-group analysis for prespecified variables included gender, age, education, heaviness of smoking, self-confidence to quit, level of social support, use of a smoking cessation aid at baseline, and frequency of assigned intervention use. No statistically significant subgroup effects were found when comparing continuous self-reported abstinence between the intervention (CTC) and control (OnRQ) conditions (see Figure 2). Although not statistically significant, higher cessation outcomes favored the OnRQ condition for participants with high school education or less (71/371, 19.1% vs 39/354, 11.1%, OR 0.53, 95% CI 0.27-1.03, *P*=.06). Similarly, higher cessation outcomes favored the OnRQ condition for those reporting high levels of social support (64/354, 18.2% vs 35/371, 9.5%, OR 0.48, 95% CI 0.22-1.06, *P*=.07; see Figure 2).

### Satisfaction and Use

As shown in Table 3, at 6 months 85.1% (359/422) of the CTC participants downloaded the app as compared with 81.8% (346/423) of OnRQ participants having downloaded or requested a printed copy of the self-help guide, (χ^2^_1(N=845)_=1.6, *P*=.23). Furthermore, OnRQ participants reported significantly higher levels of overall satisfaction (mean 3.3 [SD 1.1] vs mean 2.6 [SD 1.3]; *t*_644_=6.87, *P*<.001) and perceived helpfulness (mean 5.8 [SD 2.4] vs mean 4.3 [SD 2.6], *t*_657_=8.0, *P*<.001), as well as higher levels of frequency of use, confidence in using, ease of use, and perceptions of the intervention being well laid out as compared with the CTC participants (see Table 3).

**Table 3:** Comparison of Crush the Crave and On the Road to Quitting on use and satisfaction measures at 3 and 6 months.

Satisfaction and use			CTC^a^, n	Summary	OnRQ^b^, n	Summary	*P* value
**3 months (N=791)**							
	Downloaded		384	329 (85.7)^c^	407	325 (79.9)^c^	.03
	**Satisfaction with app, mean (SD)**						
		Used frequently	325	3.6 (1.1)	317	3.3 (1.0)	.003
		Easy to use^d^	312	2.2 (1.0)	308	2.1 (0.8)	.14
		Well laid out^d^	307	2.4 (0.9)	306	2.1 (0.7)	<.001
		Confidence in using^d^	306	2.7 (1.1)	310	2.6 (0.9)	.23
		Overall satisfaction^e^	306	2.6 (1.1)	299	3.1 (1.0)	<.001
		Overall helpfulness^f^	308	4.2 (2.6)	299	5.2 (2.3)	<.001
**6 months (N=845)**							
	Downloaded		422	359 (85.1)^c^	423	346 (81.8)^c^	.23
	**Satisfaction with app, mean (SD)**						
		Used frequently^d^	351	3.6 (1.2)	334	3.2 (1.1)	<.001
		Easy to use^d^	340	2.3 (1.1)	324	2.1 (0.8)	.01
		Well laid out^d^	337	2.5 (1.1)	324	2.1 (0.8)	<.001
		Confidence in using^d^	331	2.8 (1.1)	318	2.5 (0.9)	.002
		Overall satisfaction^e^	332	2.6 (1.3)	314	3.3 (1.1)	<.001
		Overall helpfulness^f^	337	4.3 (2.7)	322	5.8 (2.4)	<.001

^a^CTC: Crush the Crave.

^b^OnRQ: On the Road to Quitting.

^c^The values present n (%).

^d^Scale of 1 to 5: *strongly agree* to *strongly disagree.*

^e^Scale of 1 to 5: *not at all satisfied* to *very satisfied.*

^f^Scale of 1 to 10: *not at all helpful* to *very helpful.*

## Discussion

### Findings

This is the first RCT of a smoking cessation app compared with a self-help guide with a large sample size of Canadian young adults followed up at 3 and 6 months. Studies of smoking cessation apps to date have had SMS text messaging or another app as control conditions [14,24,26]. The results of this study show that there were no statistically significant differences between the intervention and control conditions on the key outcome measures. With participants lost to follow-up treated as smokers (last observation carried forward), the CTC app and OnRQ resulted in continuous abstinence rates of 7.8% (64/820) and 9.2% (72/779), respectively. Unlike this study, BinDhim et al [26] recently assessed the efficacy of an interactive smoking cessation smartphone app compared with a static information only smartphone app on adults using a double-blind RCT and found a significant difference in continuous abstinence rates of 3.2% and 7.3% for the control and intervention conditions, respectively. In comparison, our trial found a continuous abstinence rate comparable with BinDhim et al of 7.8% (64/820) for the intervention condition. In addition, Bricker et al [14] conducted a small sample size (n=196) double-blind RCT with adults to assess the efficacy of the smartphone app SmartQuit as compared with the National Cancer Institute’s QuitGuide smartphone app. The primary outcome for this smaller study was 30-day PPA at 2-month follow-up, and based on complete cases no statistically significant difference was found between the intervention (13%) and the control (8%) conditions. In comparison, our study found 30-day PPA rates at 3 months of 18% and 17% for the CTC intervention and comparison condition, respectively. Finally, Buller et al [24] conducted a very small sample size RCT with young adults (n=102) to compare a smartphone app versus an SMS text messaging app for smoking cessation. With participants lost to follow-up treated as smokers, continuous abstinence at 3 months for the smartphone app was 16% in comparison with SMS text messaging at 27%. Although substantive, this difference was not statistically significant because of the small sample.

In Canada, the typical unassisted abstinence rate based on 30-day PPA (having not smoked in the previous 30 days before being interviewed 6 months after baseline) is 5% (95% CI 4%-6%) among adult smokers based on a large sample size (n=4355) population-based longitudinal cohort study of Ontario smokers [45]. According to the same Ontario longitudinal cohort study of smokers, the unassisted 30-day PPA for young adult smokers (aged 18-29 years) was 11.5% (68/592) as assessed 6 months after baseline [46]. Similar to the findings of Ubhi et al [25], ccomparing rates of cessation with a smartphone app against population-based estimates of unaided cessation, this study also found the CTC smartphone app had favorable 6-month intention-to-treat 30-day PPA rates at 6 months compared with the unassisted abstinence rates for young adult smokers (see Table 2).

CTC was not superior to the control condition OnRQ. Rather, the primary outcome and secondary outcome measures at 6 months favored the self-help booklet control condition. However, the continuous abstinence rate and 30-day abstinence rate for CTC is comparable with previous research on smoking cessation smartphone apps [14,26] and is more favorable than what is typical among unassisted young adult smokers [46]. This study is unique in that both the CTC app and OnRQ were similar in their content and evidence base but very different in their mechanism of delivery. Furthermore, this is a large sample size study comparing a smoking cessation smartphone app with the usual self-care and low-intensity intervention of a self-help guide. This is contrary to the approach by BinDhim et al, for example, who examined 2 different apps (one was based on evidence and theory, whereas the other was not based on an evidence-informed structure). Evidence to date has demonstrated the effectiveness of mobile phone SMS text messaging interventions [20]; however, this study has revealed that although the quit rates are comparable with other studies of smoking cessation apps, an evidence-informed smoking cessation app is not superior to an evidence-informed self-help guide. These findings pave the way to examine specific evidence-informed components that do or do not translate well in the mHealth context that has implications for future research and successful scale-up [47].

It is interesting that those with higher education and those with low social support favor the CTC app and that some populations may prefer alternative low-intensity evidence-informed interventions such as self-help guides. In addition, men’s interest in using CTC is noteworthy. In our recently published qualitative work, it was found that men were particularly receptive to CTC’s ability to present personalized and relevant information in relation to their smoking behavior and engage them in autonomous behavior change [48]. That men prefer the tailoring capabilities inherent in technology-based health interventions is supported in the general mHealth literature [49]. Intervention preferences for those with perceived lower levels of social support from friends and colleagues in quitting smoking along with gender and other characteristics can be further explored.

Despite the independent and positive assessment of Ubhi et al [30] regarding user engagement and usability of CTC, findings indicate significantly higher satisfaction and perceived helpfulness with the self-help booklet OnRQ compared with the CTC app. The participants likely perceived CTC as too complex to use as compared with a self-help booklet. Therefore, there is the potential for improvements to the content and usability of CTC that may result in higher abstinence rates. This brings forward the need for qualitative research to understand user experiences and preferences in relation to an app’s design and related functions to enhance user satisfaction [48].

### Limitations

This study has several limitations. Despite a rigorous process for encouraging participants to complete follow-up, an overall response rate of 60.48% at 6-month follow-up is considered suboptimal. However, this level of follow-up response is similar to other Web-based cessation intervention studies such as BecomeAnEX with a 3-month follow-up rate of 59% [50]. Despite the less than optimal response rate, there was no differential attrition between groups as the groups were balanced with regard to all characteristics measured at baseline and at follow-up (see Table 1). As the baseline characteristics of those lost to follow-up did not differ between conditions, any possible differences in the outcome measures between conditions are unlikely to be associated with these characteristics [44]. Furthermore, the intention-to-treat principle was followed for the analysis of outcomes using 3 standard approaches to handling missing data [35,43,51].

Although it was demonstrated that the rates of continuous abstinence and 30-day PPA for the CTC intervention condition were comparable with the few trials of smoking cessation apps to date, an important limitation of this study is the lack of a no intervention control group. However, it is often difficult to avoid attrition bias when conducting trials with inactive controls [52], and inactive controls are sometimes challenged as unethical in settings in which participants could be given an existing usual care intervention [53].

Although participants were blinded, both interventions were potentially available to any participant, implying a risk of contamination. However, we took measures to minimize this through ensuring unique IPs at recruitment and only 2.2% reported use of a self-help guide and 3.2% use of a smartphone app at 6-month follow-up in groups not allocated to these interventions.

Recall bias with regard to the self-reported use of interventions is possible. Although the automated recording of the use of CTC is possible as reported elsewhere [31], it was not possible to automatically record the use of the self-help guide OnRQ and, consequently, self-reported satisfaction and use measures were chosen to allow for comparison between conditions, and there is no evidence that recall bias was different across conditions.

Finally, the lack of biochemical validation of smoking abstinence is a limitation that may have resulted in an overestimation of smoking abstinence [54]. However, a Cochrane review of low-intensity internet-based interventions for smoking cessation found that very few studies used biochemical validation given the difficulties in obtaining samples from participants [55], and expert consensus suggests that biochemical verification of abstinence is impractical and unnecessary in large studies such as this one because of cost considerations and limited face-to-face contact [56]. Furthermore, accurate estimates of the prevalence of cigarette smoking among Canadians can be derived from self-reported smoking status data [57].

### Generalizability

This study reached a large sample of Canadian male and female young adults from various ethnicities, education, and income groups, including unemployed and low-income young people, who owned smartphones and were motivated to quit smoking. The inclusion criteria of understanding, reading, and speaking English resulted in a lack of representation from young adult francophone smokers. French is the mother tongue of approximately one-fifth of the Canadian population, most of whom live in Quebec. Therefore, the study sample is limited to English-speaking Canada, and the findings may not be generalizable to young adult smokers with smartphones in other settings.

### Implications for Practice

CTC did not show a significant difference from a usual care self-help guide. Despite the rates of quitting being comparable with other smoking cessation app interventions, research into improving the overall satisfaction and helpfulness of CTC is needed before practitioners may recommend an evidence-informed mHealth app, such as CTC, to smokers willing to quit. In addition, the widespread reach that cessation apps, such as CTC, can have, particularly for hard-to-reach populations, supports the relevance and need for mHealth cessation interventions. For example, among 18- to 29-year-old people, smartphone ownership is nearing saturation among all socioeconomic groups [10], and as a result, population health practitioners need to consider the impact and the reach of these interventions as mHealth cessation interventions could potentially help to eliminate tobacco-related health disparities. Given the potential for widespread reach, effective smoking cessation apps may warrant inclusion in the overall cessation picture for Canadian young adults. Furthermore, as smartphone apps for health and healthy behavior change are so numerous and often downloaded, it is important that studies such as this are conducted and findings, particularly if not overly supportive of the effects of these apps, are published.

### Future Research

To date, the effects of smartphone apps for smoking cessation are largely unknown and this study is one of the very few trials that have been undertaken. A number of larger RCTs are underway to assess the effect of smartphone apps for smoking cessation [58-61]. In the near future, the evidence from these studies will be brought together and reviewed to determine the overall effectiveness of mHealth for smoking cessation, under what conditions and for whom. In the interim, future research to establish the cost-effectiveness of mHealth cessation interventions is needed [62]. Although the findings from this study indicate comparability with another low-intensity intervention, lower levels of satisfaction and helpfulness suggest that future research should explore the app’s usability using qualitative research [48], followed by an evaluation of the improvements and exploration of the program features or components that account for differences among smokers [63]. Due to the multicomponent nature of CTC, there is a limited understanding as to what intervention mechanisms are associated with behavior changes such as quitting smoking. Research to disentangle which elements of a multicomponent intervention are accounting for change may be useful [64]. Similar to the experience of BinDhim et al [26] testing of a smoking cessation decision-aid app, this study experienced the loss of some CTC app functionality, notably push notifications, likely because of the Apple app store and Google Play changing their regulation policies and technical specifications. Future research on smartphone apps should take into consideration these potential changing policy and technical issues. Finally, a superiority trial was conducted, and future research may consider inferiority or equivalence designs when comparing mHealth against established evidence-based low-intensity interventions such as self-help guides [65,66].

### Conclusions

CTC was feasible for delivering cessation support but was not superior to a self-help guide in helping motivated young adults to quit smoking. Both conditions in this trial are considered low-intensity self-help interventions and achieved rates of continuous abstinence comparable with other mHealth studies for smoking cessation. As smartphone apps may not serve as useful alternatives to printed self-help guides, there is a need to conduct further research to understand how smartphone apps for smoking cessation can be improved and become better in supporting population health efforts to reduce the overall prevalence of smoking.

## Appendix

Multimedia Appendix 1 Online recruitment, informed consent, and randomization.

Multimedia Appendix 2 CONSORT‐EHEALTH checklist (V 1.6.1).

## Abbreviations

CONSORT: Consolidated Standards of Reporting Trials
CTC: Crush the Crave
HSI: Heaviness of Smoking Index
ITT: intention-to-treat
NRT: nicotine replacement therapy
OR: odds ratio
OnRQ: On the Road to Quitting
PPA: point prevalence abstinence
RCT: randomized controlled trial
SMS: short message service
