# Supplementary material for: Effect of a Mobile Phone Intervention on Quitting Smoking in a Young Adult Population of Smokers: Randomized Controlled Trial
Source: JMIR Mhealth Uhealth. 2018 Oct 23;6(10):e10893. doi: 10.2196/10893 (PMC6231795; doi:10.2196/10893)
Supplement: Multimedia Appendix 1 [file mhealth_v6i10e10893_app1.pdf]

Online Recruitment, Informed Consent and Randomization

ONLINE INTRO SCRIPT:
Thank you for your interest in the quit smoking study being conducted by the Propel Centre for Population Health Impact, University of Waterloo. The study purpose is to learn what quit smoking programs are most effective for young adult smokers.

STUDY DESCRIPTION
The study has 3 parts:
Part 1: Answering questions about yourself and your smoking behaviour to see if you are eligible for the study. It should take about 2 minutes.

Next you will be asked to confirm your agreement to participate in the study. If you agree, you will be randomly assigned to receive one of two quit smoking programs. Being randomly assigned means you have an equal chance of receiving either one of the quit smoking programs. You will be asked some additional questions which should take about 10 minutes.

Part 2: Asks you to use a quit smoking program for the next 6 months to help you in your attempts to quit

Part 3: Asks you to complete a 10-minute follow-up survey in ***3 months and 6 months*** from now. We will contact you by email with a link to complete the follow-up surveys.

ARE YOU ELIGIBLE?
If you are interested in participating, click the button below to answer the eligibility questions.

**ELIGIBILITY CRITERIA**	
Q1	**In the last 30 days, how often did you smoke cigarettes? (CHECK ONE)**<br>_____ Every day (1)<br>_____ At least once a week (2)<br>_____ At least once in the last 30 days (3)<br>_____ Not at all (4)<br>[**If once/week, once/month or not at all** – Thank you for your time but our study requires participants to be daily smokers. If you would like more information on quitting smoking please visit CCS-Quit Smoking].
Q2	[IF smoking status = 1]<br>**On average , how many cigarettes do you smoke each day**<br>_____ [enter number]<br>_____ Don't know<br>_____ Refused

Q3	**When was the last time you smoked a cigarette, even a puff?** *(If you are unsure, just make your best guess).*<br>______ Today<br>______ Yesterday<br>______ In the past week<br>______ In the past month<br>______ Don't know<br>______ Refused
Q4	**Do you intend to quit smoking within the next 30 days?**<br>______ Yes<br>______ No (INELIGIBLE - Skip to 'ineligible' script)<br>______ Don't Know/Can't say (Skip to 'ineligible' script')<br>[**If NO or Don't Know/Can't Say** – Thank you for your time but since this study involves quitting smoking, we require individuals who would be willing to quit smoking in the next month. If you would like more information on quitting smoking please visit CCS-Quit Smoking].
Q5	**What is your date of birth? ____ ____ / ____ ____ (mm/yyyy)**<br>We require this information to determine if participants are between the ages of 19-29, and therefore eligible for our study. This study is only suitable for young adult smokers.<br>[**If not between 19-29** – Thank you for your time but this study is only suitable for young adults between the ages of 18-29. If you would like more information on quitting smoking please visit CCS-Quit Smoking].
Q6	**Are you comfortable understanding, reading, and speaking English?**<br>______ Yes<br>______ No<br>[**If NO** – Thank you for your time but this study requires that participants are able to fluently understand and speak English. If you would like more information on quitting smoking please visit CCS-Quit Smoking].
Q7	**Do you currently own and use an Android or iPhone smartphone or tablet?**<br>______ Yes<br>______ No<br>[**If NO** – Thank you for your time but this study requires that participants own an Android or iPhone smartphone or tablet for the purposes of being contacted by the research team. If you would like more information on quitting smoking please visit CCS-Quit Smoking].

Q8	**Are you aware of anyone in your household (besides yourself) who is participating in this study?**<br>______ Yes<br>______ No<br>______ Don't know/can't say<br>[**If YES or Don't Know/Can't Say** – Thank you for your time but this study requires that only one individual per household participate in the study. If you would like more information on quitting smoking please visit CCS-Quit Smoking]
Q9	**Were you referred to this website by a friend who is already participating in this study?**<br>______ Yes<br>______ No<br>______ Don't know/can't say<br>[**If YES or Don't Know Can't Say** – Thank you for your time but this study requires individual participants who are not acquainted with one another and who have not been referred to the study website. If you would like more information on quitting smoking please visit CCS-Quit Smoking]
**ELIGIBILITY – YES**<br>**You are eligible to participate**. The information below will help you decide if you want to participate.	
**CONSENT PROCESS**	
Participation is voluntary. You may stop using the program or withdraw at any time by contacting the research team. If you withdraw, your data will be destroyed.<br><br>To thank you for participating you will receive **$35**; **$10** now for enrolling in the study, **$10** for completing the 3 month follow-up survey, and **$15** for completing 6 month follow-up survey. In addition, your name will be entered into a draw at the end of the study (Spring 2015), for an **iPad 2 Air 64GB**.<br><br>Responses are confidential. Only the research team will see your answers. Data is grouped; no individual is identified. Names, emails, and addresses are only used to contact you for the follow-up survey and to mail you $35, and to enter you name into the draw.<br><br>Ethics clearance has been given by a University of Waterloo Research Ethics Committee. Contact … if you have concerns.<br><br>**Questions about the study?**<br>Contact: …<br><br>**I understand the information and that by clicking YES, I agree to participate in the research study**	

and accept the terms as they are outlined above.

_____ YES

_____ NO

Randomization

Please provide your email address and a telephone number that you would like us to use to contact you about the follow up survey 6 months from now. ***Please remember that your email and other contact information are used by research staff only to contact you for the follow-up surveys.***

Email address: ____

Telephone: [________] [________] [_______________]

____ REFUSE

[Script: *Thank you but if you are unwilling to provide an email address you will not be eligible to participate in the study. We require your email address so that we may contact you for the follow up survey.]*

Please provide your address information so that we can mail you the $10 honorarium for enrolling in the study. You will receive **$10** for completing the follow-up survey in 3 months and $15 for completing the final survey 6 months from now. In addition, your name will be entered into a draw at the end of the study for an **iPad Air 2 64GB.**

Please remember that your name and other contact information are used by research staff only to send you the cheques and enter you name into the draw.

Full Name:

Street Address (include Apt. or Unit #) : ______________________________

City/Town: ____

Province: ____

Postal Code: ____

Thank you for enrolling in the study. Please click the 'Save and Continue' to finalize your registration for the research study and to receive your quit smoking program. **[randomize to either 1 or 2]**

1. **On the Road to Quitting Self-Help Guide – Description**

At the end of the survey we will provide you with a link to download a copy of the **On the Road to Quitting** Self-Help guide for quitting smoking available from Health Canada. We will also email you a copy or you may request a hard copy of the guide.

In order to participate in the study, you must use the **On the Road to Quitting** guide over the

next six months to help you quit smoking. At 3 and 6 months, we will follow up with you to ask about your smoking and quitting behaviour and to collect some information about the quit smoking program that you received.

2. **Crush the Crave Quit Smoking Smartphone App – Description**

At the end of the survey we will provide you with a link to download the **Crush the Crave** quit smoking smartphone app to help you quit smoking. This link will allow you to download the Android or iPhone app from either the Google Play or iTunes App Store depending on your phone's operating system.

In order to participate in the study, you must use the **Crush the Crave** smartphone app over the next six months to help you quit smoking. At 3 and 6 months, we will follow up with you to ask about your smoking and quitting behaviour and to collect some information about the quit smoking program that you received.
